# Experiences of receiving a bystander intervention during a suicide attempt on the railways

**DOI:** 10.1192/bjo.2026.10991

**Published:** 2026-03-05

**Authors:** Bethany Cliffe, Dafni Katsampa, Jay-Marie Mackenzie, Ian Marsh, Lisa Marzano

**Affiliations:** Psychology, University of Westminster, London, UK; Psychology, Middlesex University, London, UK; Allied Health, Canterbury Christ Church University, Canterbury, UK

**Keywords:** Suicide prevention, bystander intervention, railway suicides

## Abstract

**Background:**

Promoting bystander interventions is a core strategy to prevent suicides in public places, but little is known about how people with lived experience perceive this intervention.

**Aims:**

To explore people’s experiences of receiving an intervention during a suicide attempt on the railways.

**Method:**

Interviews were conducted with 28 people with experience of receiving a bystander intervention during a suicide attempt on the railways. Interview data were triangulated with an online ethnography exploring posts made on forums and similar platforms openly discussing suicide.

**Results:**

Using reflexive thematic analysis, five themes were generated: (a) ‘I’m a good actor… we all carry a mask’: concealing feelings and intentions; (b) ‘It kind of draws your attention away but it doesn’t almost shine a spotlight’: interventions should be gentle and not draw more attention; (c) ‘People that were getting in my way were just making me want to try harder’: interventions can trigger difficult feelings in the moment; (d) ‘I did feel different, I felt better’: the power of small talk; and (e) ‘You feel like a human being and you feel like they actually care’: wanting to feel cared for.

**Conclusions:**

Findings suggested that some people actively avoid receiving an intervention by concealing how they are feeling in that moment. Interventions that are gentle and do not draw attention may be preferable, such as making small talk and simple gestures such as smiling or being near the individual. Conversely, more intrusive or aggressive interventions may be triggering and exacerbate suicidality.

In 2023, the rates of suicide were the highest they have been since 1999,^1^ and between 2023 and 2024, 276 suspected suicides took place on the overground rail network.^2^ This accounts for roughly 4% of all suicides in the UK.^3^ One of the core strategies for suicide prevention in public places is promoting an intervention by a third party,^4^ also known as ‘bystander intervention’, which can range from verbal to more physical interventions such as restraint or restricting the individual’s access to a dangerous location. These may be initiated by the general public, members of staff, emergency services or people known to the individual.

## Bystander interventions on the railways

Analysis of railway incident data from New South Wales, Australia, identified that 22% of 635 successful suicide interventions were instigated by bystanders. This was either through direct intervention (11%) or raising the alarm (11%).^5^ This highlights opportunities for bystander interventions, which is in accordance with the ‘Small Talk Saves Lives’ campaign that encourages the public to engage with anyone who appears to be in distress;^6^ following the campaign, 74% who had seen it said they would approach someone in distress.^7^ Research investigating effective interventions underscores the importance of breaking the dissociative state the individual may be in, helping them to feel safe and connected, moving them to a safer location and summoning help.^8^ These findings challenged potential misconceptions around how people having thoughts of suicide may present. For example, they were often described by themselves and others as ‘numb’ and ‘zoned out’, rather than visibly in distress.^8^ This resonates with broader research around how people act before a suicide related incident,^9^ and highlights the importance of learning more about this experience of feeling suicidal in a public place and receiving an intervention. Mackenzie et al.^10^ found examples of unhelpful action included bystanders being rude or making a scene and drawing more attention to the individual.

## Aims

It is important to learn more about positive and negative elements of public interventions to inform public awareness campaigns and possible suicide prevention training for bystanders. There is limited research around the experiences of those receiving an intervention, and the research that has been done has begun to challenge misconceptions around how people feeling suicidal may feel or respond to an intervention in that moment. The current study aimed to address this by exploring the perspectives of those who have attended the railways with suicidal thoughts or intent and received an intervention.

## Method

This paper combines findings from qualitative interviews and a scoping online ethnography to gain insight into how people experience receiving an intervention on the railways.

### Qualitative interviews

Participants were recruited from an online survey exploring individuals’ perspectives on receiving an intervention when experiencing suicidal ideation, or of intervening when somebody else appeared to be experiencing suicidal ideation (findings in relation to the latter are presented separately,^11^ as are other findings from the survey,^10^ and the data reported here have not been analysed as part of any other paper). The survey took place in 2019 and was advertised widely on social media, the Samaritans website and through leaflets/posters on university bulletin boards. A total of 2270 people responded to the survey, and 240 of those had experienced suicidal thoughts at railway locations. Within the survey they were asked if anyone intervened in any way, including approaching and asking if they were okay or making small talk, non-verbal gestures (e.g. eye contact, smiling), calling for help, preventing access to means of suicide, restraining or other. From this group of 240 respondents, 39 individuals indicated that someone had intervened or interrupted them and, on this basis, were invited to participate in a follow-up interview (provided that they had not been experiencing suicidal thoughts or attempts in the past month, and were aged 18 years or above). Twenty-one respondents agreed to participate in an interview.

Interviews were semi-structured, and an interview schedule was developed to address the research question (see Supplementary Material available at https://doi.org/10.1192/bjo.2026.10991). Topics included their experiences of suicidal ideation surrounding the railways, experiences of being distracted/receiving an intervention when having suicidal thoughts, and what they thought makes for a ‘good’ intervention to prevent suicides on the railways. Depending on participants’ preferences, interviews took place either online over Skype (for Windows; www.skype.com) (*n* = 14), or in person at Middlesex university or local Samaritans branches (*n* = 7). Interviews were recorded and then transcribed verbatim.

Participants were aged 20–58 years (mean age 35 years). Thirteen participants identified as women and eight identified as men. Eighteen participants were in employment, two were unemployed or receiving state benefits, and one was retired. Interviews lasted between 35 and 89 min (mean, s.d. = 14.6), and all were conducted by the same researcher (D.K.).

### Online ethnography

Data were collected from posts made in online forums or platforms that are used to openly discuss suicide, between 14 December 2018 and 14 December 2019, to roughly mirror the time period in which interviews were taking place. Online interactions were observed in publicly available moderated spaces that are widely used, as indicated by the number of active users across the sites. Search terms relative to suicide, intervention and the railways were used and, after excluding data that was not relevant to the research question, this resulted in 55 discussion threads on one online forum, 199 posts with 1228 associated comments on another and 872 on another. Given the anonymity of these sites, it is not possible to know the demographics of those making posts/comments. Field notes were made throughout data collection. Other insights from this analysis are presented elsewhere.^10^

### Ethical approval

The authors assert that all procedures contributing to this work comply with the ethical standards of the relevant national and institutional committees on human experimentation and with the Helsinki Declaration of 1975, as revised in 2013. All procedures involving human patients were approved by the Psychology Research Ethics Committee at Middlesex University (reference number ST019-2015). An advisory group of people with lived/living experience of suicidality was involved in developing the research materials, and the Samaritans were also consulted. Written consent was obtained from all interview participants. All interview transcripts were anonymised, and to protect the privacy of online forum users, no website names or direct quotes have been used. All online comments have been paraphrased to illustrate the key findings.

### Data analysis

The data from both studies were analysed separately and then triangulated.

The interview transcripts were analysed with reflexive thematic analysis, following the six steps outlined by Braun and Clarke.^12–14^ First, the transcripts were read to ensure familiarisation. The transcripts were then coded following an open and inductive approach. Codes and relevant quotes were extracted into a table and developed into themes. This was an iterative process whereby mind maps were created to explore different organisations of codes, and these were reviewed alongside the data and discussed with the research team.

The online ethnography data were also analysed with a thematic approach, to understand patterns of meaning within the data and field notes. Coding was done through an iterative process of open, axial and selective coding. First, each post was coded in a way that captured its key thoughts and ideas. Second, the posts were then reviewed (axial coding), and broader themes were developed to consolidate the open codes from the first stage.

After both sets of data were analysed separately, the findings were integrated by using a ‘following a thread’ approach.^15^ In this way, a theme from one data-set was chosen and then ‘followed’ across to the other to create a holistic understanding of the findings. This resulted in five key themes outlined below and direct quotations are used with each participant being assigned a number, aside from online forum posts which are paraphrased quotations and denoted in text as being an ‘online forum user’.

## Results

Interventions primarily took place at railway locations across the UK, but other examples were given of interventions received on top of a multi-storey car park and a bridge. For the purpose of this analysis, only insights relating to railways have been included. Participants described receiving interventions from people they knew such as friends or family members, emergency services and strangers.

Five themes were developed: (a) ‘I’m a good actor… we all carry a mask’: concealing feelings and intentions; (b) ‘It kind of draws your attention away but it doesn’t almost shine a spotlight’: interventions should be gentle and not draw more attention; (c) ‘People that were getting in my way were just making me want to try harder’: interventions can trigger difficult feelings in the moment; (d) ‘I did feel different, I felt better’: the power of small talk; and (e) ‘You feel like a human being and you feel like they actually care’: wanting to feel cared for.

### Theme 1: ‘I’m a good actor… we all carry a mask’: concealing feelings and intentions

Some participants simply did not expect to receive an intervention, assuming that others *‘*would just kind of walk by’,^5^ whereas other participants actively went out of their way to avoid intervention. This included, for example, visiting the station when they assumed others would not be there:

‘I probably would have waited until there was actually nobody about, so … which does happen at that station, it’s very quiet.’^14^

This was also noted in the online ethnography, where some posts advised against going to the railways to die by suicide because of the possibility of intervention. There were also discussions around the best time of day to go, what to wear and how to act, with some having scouted out locations beforehand. Participants in the interviews also discussed steps taken to avoid an intervention, which included participants lying about their feelings to prevent concern from others:

‘I have hidden it before where I’ve felt really low and I just wanted to like kill myself, this lady, bless her, stopped me in the middle of the road and she was like, are you OK? And I had like a red face, I’d been crying, ehm … I had like … just my facial expression was obviously not right, and I was like, no, no, I’m fine, I’m fine, and that was it. And she just went, are you sure? I was like, yeah, yeah, yeah, I was like, honestly it’s fine, just leave me, I’m fine, and I just walked off.’^10^

However, for some this was not just about not wanting to be stopped from attempting suicide, but it was also out of concern for those around them. Interview participants reported fears of others getting injured trying to save them:

‘They might well have hurt themselves or even killed themselves trying to save me, which I would, certainly wouldn’t want.’^14^

They also discussed being concerned about upsetting the person intervening, and online forum users urged others to ‘think about the trauma for others involved’. Findings from both datasets showed that some people prioritised bystanders’ feelings over and above their own distress and current suicidal ideation:

‘I’d kind of become more worried about upsetting her than how I was feeling. […] Ehm … again it’s kind of difficult to explain. One of the major problems with my depression over the years is that I keep it all to prevent other people from feeling how I felt … or for fear of upsetting someone and making them feel … feel down. So when she’d asked me how I was feeling, my instinct was just to say, yeah I’m not feeling great but I’m OK.’^3^

In addition to lying about their feelings, this reluctance to possibly upset strangers around them also resulted in some participants altering their outward behaviour and demeanour to convey a sense of wellness:

‘I’m a good actor, we all … we all carry a mask ehm and eh I was just being a […] (sighs) you know you put your … you put your shoulders back and carry on.’^16^

One participant referred to this concept of wearing a ‘mask’ to hide their feelings as an everyday practice, as opposed to something that is unique to a suicide attempt. This suggests it is part of their routine for ‘surviving’ that enables them to avoid people seeing their true feelings:

*‘*There’s only been a handful of times I think where I’ve like properly lost it in a public place and you can tell! But the rest of the times it’s like nobody would know because you just have that mask on, that everyday surviving mask that people can’t see through.’^7^

### Theme 2: ‘It kind of draws your attention away but it doesn’t almost shine a spotlight’: interventions should be gentle and not draw more attention

In some instances, it was not always possible to avoid intervention, and for some participants, an intervention was welcomed. However, participants had clear ideas about what an intervention should look like. One aspect of a good intervention that underscored all others was the need for it to be ‘light touch’ and not too intrusive or direct:

‘Personally, I would say a more light touch, rather than going heavy because … it’s … it’s something, it breaks the thought […] it kind of draws your attention away but it doesn’t almost shine a spotlight on, oh this person’s about to do this.’^4^

As this participant highlights, a light touch intervention is enough to distract them without drawing more attention to the situation, which could be overwhelming and make things worse:

‘I think it’s like, for me, it would be not creating a scene […] I think the fewer people you can have involved, the better, because otherwise that could just be overwhelming in itself, that you’ve now got six, seven, I don’t know, twenty people watching you or asking questions or … ehm, trying to get involved. And nine times out of ten people are trying to help but sometimes … trying to help too much can have the adverse effect.’^7^

This might be particularly challenging at railway stations given that they are public so it may ‘cause a lot of embarrassment’.^15^ Similarly, participants described worries about being ‘outed’, and not necessarily wanting people to know what they are feeling:

‘There’s something about being just friendly and not necessarily really direct, you know, it is kind of like, oh this weather, how are you doing? Not like, oh my God you look really sad, or oh my God I’ve noticed all these cuts on your arm, are you alright?! That’s just not helpful! […] And it kind of feels also that someone’s outing you, you know, you don’t always want someone to know that you feel that way.’^5^

It was suggested that a preferable alternative could be a ‘stepped’ approach which starts with a simple gesture of kindness to encourage the individual to engage:

‘Well I’d imagine it would be in a sort of stepped process. So sort of try to make eye contact and make a warm gesture, like a smile. That person opens up in some way and looks more engaged with you then you try and make contact and approach them, but do it very slowly and steadily.’^15^

Similarly, it was acknowledged that an intervention should not be forced upon someone, and that certain types of intervention may exacerbate the situation if, for example, emergency services were to arrive:

‘If I didn’t want an ambulance to turn up, that could be the reason that would push me over the edge, because … I don’t know, one of my worst fears is being sectioned, and that’s the last thing is … would be to dragged away by ambulance crew or kind of create that kind of scene! […] sometimes there’s nothing worse than being forced into something that you don’t want to happen.’^7^

‘I’d been sat up, just my legs dangling over the edge and apparently several members of the public had called them and I was chatting to them. And … out of the blue, I didn’t even know it was coming, two very large officers grabbed me from behind to sort of pull me back over.’^3^

Online forum users also discussed being *‘*pulled’ away from the platform edge, and some interview participants even described being left with injuries following interventions that were aggressive and where physical force had been used to remove them from the station:

‘I literally got picked up and physically dragged across the platform out … he [ex-boyfriend] wasn’t very nice […] I had bruises on both my arms, I had … I think I cracked a rib but I didn’t go to hospital, but every time I breathed for like a few weeks it was really hurting. I was, yeah, covered in bruises.’^10^

### Theme 3: ‘People that were getting in my way were just making me want to try harder*’:* interventions can trigger difficult feelings in the moment

Some participants described difficult or worsened feelings that resulted from their life being saved, particularly those who still lived with suicidal thoughts and were not happy to have been prevented from dying. Online forum users expressed distress around the opportunity having been ‘taken away’ from them and felt that ‘people should mind their own business, what right do people have to stop someone?’, and ‘it should be illegal to intervene’.

For interview participants, their distress at having been prevented from dying was also influenced by their experience of the intervention as, for those who had received poor treatment, this seemed to feed into their continued suicidality:

‘I don’t understand why they [the police] won’t just let me go […] I know I’m a waste of time, they try and tell me I’m not, but then they arrest me for like being a public nuisance, so I clearly am, so why don’t they just let me do it?’^13^

In some instances, the experience of having their life saved through intervention increased their determination to find another way of dying by suicide, particularly methods that offered less opportunity for intervention:

‘Because at that moment in time, I was pretty single-minded in what I wanted to do. And people that were getting in my way were just making me wanting to try harder.’^17^

Some felt more positive about the intervention looking back. They were able to acknowledge feelings of gratitude and relief after the intervention despite feeling angry or annoyed during:

‘When I first answered the phone on my mobile, I went what?! Because I were contemplating doing it, and then he said oh it’s [friend’s name] like, and that’s the intervention that stopped me doing it. Yeah, I were mad that he’d rang at the point, but after I’d been speaking to him I weren’t mad because he stopped me doing it.’^18^

### Theme 4: ‘I did feel different, I felt better’: the power of small talk

Clearly, interventions can sometimes have negative consequences, particularly those that are intrusive or even hostile. When discussing what makes a ‘good’ intervention, small talk was often referenced as a simple yet effective tool for disrupting suicidal thoughts, helping them to feel less alone and improving their mood:

‘It was just general things of you know what I like doing, ehm the things we enjoyed about the lane, the pollens(?), me telling her [a stranger] about things that I’d got up to sort of years before when I was younger and … And it did make a difference. By the time we’d walked up the lane and got back down to the other end of the lane and she needed to go on her way, I did feel different, I felt better.’^3^

This was also expressed by some online forum users who recounted having been saved by bystanders who had talked to them and held their hand to help them through the difficult situation. Interview participants also spoke of the longer-term impact that small talk had had for them, such as changing their thought process to focus more on other, more positive areas of life:

‘She [a stranger] just started like talking to me about you know like as I say a holiday she’d had and ehm what a nice time she’d had with her family and … you know, ehm it’s nice to look forward to things and ehm you know and it just sort of … because my mind was sort of fixed on one thing, it changed … you know it’s like CBT [cognitive–behavioural therapy] or whatever you know, it just changed your thought pattern and you think … oh yeah, there are other things to think about and you know life isn’t always dark and […] well it sort of relaxed me and changed my focus.’^19^

Following on from the earlier theme about interventions needing to be ‘light-touch’, the gentle and calm nature of those engaging in small talk was noted as a key factor in it being effective:

‘But they did it gently, they weren’t like in my face.’^19^

‘The small talk and the calmness of the lady that spoke to me that particular day, was really helpful.’^3^

However, an issue raised by participants was that small talk is not always comfortable within British culture, and people may be reluctant to engage with strangers:

‘To me, growing up, it was always a … if someone talks to you and you don’t know them, they’re strange, avoid them. It’s like, it’s seen as a sort of a … go away kind of thing. Whereas that doesn’t seem to be the case everywhere. So kind of getting through the British culture might be an issue, which is a challenge. So you know encouraging British people to talk to strangers about nothing is an issue, it’s a barrier.’^11^

It may be that different approaches are needed in different settings. Nevertheless, it seemed as though the actual content of the conversation was not as important as the fact the conversation was happening:

‘So that could be about the trains, it could be about the weather, it could be about the football that was on last night, it could be about the … that’s a really nice coat you’re wearing, it really doesn’t make the slightest bit of difference, it’s not what you talk about, it’s the fact that you have the conversation’ (Participant 8).

### Theme 5: ‘You feel like a human being and you feel like they actually care’: wanting to feel cared for

It seemed as though the most important part of an intervention was being listened to, being treated like a human being and feeling cared for:

‘My husband now, he’s like come in and found me like … really like distressed and things like that, and he’s sat down and talked to me and said, right, let’s talk about this, what can we do, what can I do, what can we do together kind of thing. And I think that is what people … in my mind just need to be a bit more aware of, that that is probably the better way to … well that is the better way to go for most people. Ehm, because you feel like a human being and you feel like they actually care.’^10^

For some participants this was about receiving reassurance and words of comfort, but for others they felt cared for when they received practical support in that moment. This included helping them to get home safely, thinking for them when they are unable to think for themselves, and just staying with the person whilst they are in a vulnerable position:

‘When I can’t even hear myself think for what’s going on in my head, to have someone […] in essence thinking for me because I can’t do that right in that moment.’^7^

The fact that people cared enough to engage left participants feeling reassured and as though their faith in humanity had been restored:

‘But I guess in a way the chat I had with that woman [a stranger] kind of made me, sort of gave me a little bit more faith in humanity, you know, yeah OK the people I was surrounded by weren’t very good but you know there is at least one other good person out there who’s willing to say hi and you know talk about the horses in the field, you know?’^3^

Nevertheless, although some participants appreciated feeling cared for in the moment, there was an acknowledgement that longer-term support is necessary to resolve some of the issues triggering the suicidality and avoid being in the same situation again:

‘I’d like an intervention to happen if something was put in place to sort out like the problems in my life, so it stops, so it gets better and … and I’m not so ill all the time. But if there’s an intervention when I’ve had eh mental health help or crisis team stuff like that, they help you to the point where you’re not feeling suicidal at that moment, and then you’re still in the same situation.’^20^

## Discussion

This study explored people’s experiences of receiving an intervention when having suicidal thoughts/intent on the railways. The findings highlight the variation of experiences that people have had, with the more negative interventions seeming very intrusive, confronting and sometimes even aggressive. Negatively experienced interventions resulted with participants feeling embarrassed and helpless, which fed back into their suicidality and determination to die. The more positive experiences stemmed from more gentle or subtle interventions that involved kind gestures or light-hearted small talk, that left participants feeling cared for, reassured and listened to.

It was clear from some participants that they did not expect or want to receive an intervention, and some actively tried to avoid it. Concealment of suicidal feelings to avoid intervention is widely reported within the literature, with reasons for this including wanting to remain in control, wanting to avoid being ‘talked out of it’, and fearing hospital admission.^9,17,18,20,21^ This mirrors the current findings, in which participants were anxious about the presence of emergency services and people ‘interfering’ with their intention to die. However, this avoidance of intervention also stemmed from concern for others’ well-being. There is evidence to suggest that bystanders can indeed be negatively affected from intervening; interveners have reported struggling with not knowing the outcome for that individual, and feeling some lasting trauma from the experience.^8,11^ Consequently, there is a clear need to ensure that those who may witness or intervene in a suicide attempt have their own support following the incident.

Previous research has focused on the process of interventions and what makes them effective,^8,10,11^ but participants in the current study also discussed the more negative aspects of interventions. They emphasised that interventions which were confronting or drew more attention could be detrimental to their well-being and exacerbate their suicidal thoughts in the longer term. The current study extends Owen’s et al’s work by showing that interventions are not one-off events, but can have lasting emotional and relational consequences for both the suicidal person and the intervener.^8^ These findings underscore the importance of bystander training programmes to equip the public with skills in suicide prevention. Organisations such as Samaritans and the Zero Suicide Alliance run such training courses,^16,19^ and research has found that they can increase both knowledge and confidence for bystanders intervening.^22^ Training such as this could therefore mean that less people are exposed to harmful bystander interventions during a suicide attempt.

It is known that a suicide attempt is a significant risk factor for further suicide attempts,^23^ and participants in the current study reported that harmful interventions increased their suicidal thoughts and, in some cases, made them more determined to die by suicide. Consequently, it may be that those who receive harmful or physical interventions may be at even greater risk of subsequent suicide attempts and may be less likely to seek help in the future. There seems to be a tension between the imperative to save lives, and individuals not wanting to be saved in that moment. Physical interventions may indeed be ‘necessary’ in emergency situations, research from New South Wales, Australia, found that 85% of bystander interventions were physical, 77% of which were deemed ‘heavy’, such as tackling or restraining.^5^ Nevertheless, it is important to be aware that this can make the situation worse in that moment and so follow-up care is necessary to ensure the individual receives support around their suicidal thoughts and what triggers them.^24^

In addition to negative aspects of interventions, participants also shared what has worked particularly well in keeping them safe. Previously, Owens et al investigated what makes ‘successful’ interventions from the perspectives of those intervening, which identified that small talk about everyday things can help to ‘burst the bubble’ and bring the individual back to the present.^8^ These findings were echoed in the current study, where participants highlighted the potential that small talk about the weather or holidays could have for disrupting suicidal thoughts. Even small gestures such as smiling and eye contact were noted by participants as effective ways of engaging someone, as also noted by people who have intervened to prevent suicide at a railway location.^11^ This supports the role that the general public can play in suicide prevention using light-touch, gentle tactics, without necessarily having any prior skills or training, as is endorsed by campaigns such as ‘Small Talk Saves Lives’.^6^

### Strengths and limitations

This study adds to the limited literature exploring the perspectives of those who have received an intervention on the railways during a suicide attempt. Triangulating from two data sources also diversifies the experiences represented here, and incorporating data from online forums may capture perspectives from those who may be less inclined to take part in interview-based research. There are clear implications from this study around what makes a good intervention and what may make someone’s suicidal feelings worse, which can inform the development of future training and guidance.

There are also some limitations to acknowledge. First, the interviews in this study focused on people’s experiences at UK railways and therefore may not be applicable to other settings where people die by suicide, such as bridges or railways outside of the UK. Similarly, the railways in the UK are very diverse, including the overground, the underground, level crossings and so on, and it may be that the experiences represented here do not apply to the whole railway network. Nevertheless, there was diversity amongst the sample in the types of interventions they had received, including from people they knew, emergency services and strangers. Future research should seek to explore perspectives of intervention in other contexts.

## Supporting information

Cliffe et al. supplementary material Cliffe et al. supplementary material

## Data Availability

Data is not available in order to protect participants’ anonymity.

## Supplementary material

The supplementary material is available online at https://doi.org/10.1192/bjo.2026.10991
