# Supplementary material for: Experiences of receiving a bystander intervention during a suicide attempt on the railways
Source: BJPsych Open. 2026 Mar 5;12(2):e79. doi: 10.1192/bjo.2026.10991 (PMC13107300; doi:10.1192/bjo.2026.10991)
Supplement: Cliffe et al. supplementary material [file S2056472426109910sup001.docx]

**Interview Guide for Participants with lived experience of suicidality on the railways**

**(Individuals who made a suicide attempt, or came very close to doing so, on the railways)**

**1. Can you tell me a bit about yourself…**

**Socio-demographic details:**

- Age
- Gender
- Education; employment; family etc [interviewer to encourage free narrative, but use the Oxford Monitoring checklist to ensure that key information is covered]
- Any history of mental health problems; psychiatric treatment; hospitalisation

*One of the key aims of this research is to inform initiatives to prevent suicide specifically on the railways, so I have some questions about your thoughts and experiences in relation to attempting suicide in this context, if that’s ok.*

**2. Could you tell me in your own words about the thoughts you have had about attempting to harm yourself or end your life on the railways?**

**How many times have you experienced such thoughts?** How frequently? How long have you experienced/been experiencing these thoughts for? When was the most recent time you had had such thoughts? Etc.

**Why this particular method (as opposed to other methods of suicide)?** (Prompt about other methods considered and/or used in the past or since. How do these compare to railway suicide?)

**Do you know (of) anyone else who has attempted or died by suicide using this method?** (Prompt if applicable: Can you tell me a bit more about that please? What happened? Who was it? Etc.)

**Did anything in particular trigger your thoughts about attempting suicide using this method?** (Prompt: e.g. did you hear or read about specific events online, or in the news? Station/train announcements or other information?)

**Can you tell me about your thoughts of dying in relation to this method? (**What did you think were the chances you would die using this method?)

**Did you research this method?** (How did you go about researching this method (and/vs. other methods)? Did this include online searches? What sort of site/sites did you research? How did you find/search for these? What sort of information did you find out? What role did this play in encouraging/discouraging you from using this method? Did you take part in, or read, any online discussions about using this and/or other methods of suicide? What were the effects of these? Etc.)

How intensely have you experienced thoughts of suicide on the railways? **Did you have a specific plan?** Were these thoughts impulsive/intrusive? Did these thoughts involve specific locations or times? (If applicable, why the tube as opposed to a main railway line? Etc. )

**Can you tell me a bit more about the time, or times, when you acted on these thoughts, or came close to doing so?** (e.g. when/how many times did this happen? When? Where were you? Why that specific time/location? Did you have a specific plan? Were you injured? What happened afterwards? Etc.)

**What (might have) prevented/dissuaded you from acting on these thoughts?** Did you discuss them with anyone?

**3. As part of this research, we are exploring people’s experiences and views about being interrupted or distracted – by someone or something – when suicidal on the railways, whether or not that intervention might have been helpful**

**Did this happen to you at any point?** If so, can you tell us a bit more about what happened (ask about a specific event, with the option to the recount more)? Who/what intervened? How? What did they say/do? What was helpful/unhelpful? What was the immediate effect of that? (e.g. did it stop/delay/postpone/move your attempt? Did it make things worse in any way?) Was anybody else present? (What did they do? Etc.) Looking back, did that affect you in any other way? How do you feel about this ‘intervention’ now?

**Did you have any expectations that this might/might not happen?** Was anybody (else) present when you attempted/considered suicide on the railways? Was this deliberate? If applicable, how did they react? If nobody intervened, did you get a sense that anybody was aware of what was happening/how you were feeling? What might have given this away? Did you try and conceal how you were feeling/what you were planning in any way? Would you have liked someone to intervene? Who? How?

**What are you thoughts on encouraging people to make interventions in these sorts of situations/locations?** (Are there any potential challenges, difficulties or potential to make things worse? If so, how could this be avoided?)

**Have you ever been in this situation yourself (e.g. in relation to someone else who might have been at risk of suicide)?**

**What makes for a safe and helpful intervention?** What message would you give based on your experience, to people intervening when someone around them is suicidal?

**Do you have any other experiences of this, e.g. in other contexts?**

**4. Is there anything that you think Network Rail and other people could do to prevent suicide attempts on the railways?**

What (more) do you think the rail industry could do to help prevent suicide on the railways?

**What/who else could help?**

• Improvements to railways (re-design of railways, stations?)

• Barriers to attempts? Observation? Bystanders? Transport police?

• Interventions, support (e.g Samaritans posters, phones, volunteers)

**Is there anything that could make things worse?** (Impact of media reporting; station/train announcements)

**Are you aware of any of the initiatives already in place to help prevent suicide on the railways** (e.g. Samaritan volunteers/posters at stations etc.)? What do you think about these? Are you familiar with Samaritans’ *Small Talk Saves Lives* campaign? If so, did this influence your behaviour/thoughts about this in any way? Etc)

**5.** **It’s been great talking to you, and we really appreciate your help with this. Is there anything that you would like to add or ask me about the project?**
